# A Retrospective Cohort Study of an Outbreak of Cryptosporidiosis among Veterinary Students

**DOI:** 10.3390/vetsci4020029

**Published:** 2017-05-24

**Authors:** Jackie Benschop, Christina M. Booker, Tui Shadbolt, Jenny F. Weston

**Affiliations:** 1 Molecular Epidemiology and Veterinary Public Health Laboratory, Institute of Veterinary, Animal & Biomedical Sciences, Massey University, Private Bag, Palmerston North 4442, New Zealand; 2 Institute of Veterinary, Animal & Biomedical Sciences, Massey University, Private Bag, Palmerston North 4442, New Zealand; christinabooker@hotmail.com (C.M.B.); J.F.Weston@massey.ac.nz (J.F.W.); 3 MidCentral Public Health Service, Palmerston North Hospital, Palmerston North 4440, New Zealand; Tui.Shadbolt@mpi.govt.nz

**Keywords:** cryptosporidiosis, calves, veterinary students, outbreak, zoonoses

## Abstract

An outbreak of gastrointestinal illness occurred among a cohort of 56 veterinary technology and 100 veterinary science students at Massey University over an eight-week period in 2013. This coincided with calving in New Zealand’s seasonal dairy farming system and a time when calves with diarrhoea are commonly seen by veterinarians. Laboratory and epidemiological investigations were instigated by MidCentral Public Health Service (MCPHS) in conjunction with the Institute of Veterinary, Animal and Biomedical Sciences (IVABS) at Massey University. Eighty students responded to a questionnaire of which 19 met the case definition, a 24% attack rate. Faecal specimens from seven students contained *Cryptosporidium* oocysts and *Cryptosporidium parvum IIa* A18G3R1 was identified from one of the specimens. The inferred median incubation period was five days (range 1–12 days). All of the cases were self-limiting, characterized by diarrhoea, abdominal cramps, and in some cases vomiting, headache, and fever. Having contact with calves with diarrhoea was significantly associated with increased adjusted odds of being a case (OR 10.61, 95% CI 1.87–108.29 for one week of contact; OR 55.05, 95% CI 3.80–1931.18 for two weeks of contact). Outbreaks of cryptosporidiosis had occurred previously among veterinary students at Massey University, but the extremely high infectivity of *C. parvum* resulted in student illness despite enhanced hygiene precautions.

## 1. Introduction 

Cryptosporidiosis is a common gastrointestinal disease caused by a pathogenic protozoan, of the genus *Cryptosporidium* (Apicomplexa: Cryptosporidiidae) [1], which affects all mammals, birds, reptiles, and possibly fish [2] New Zealand health authorities reported 19.7 cases per 100,000 between 2004 and 2011, although there were significant regional differences that correlated to exposure to livestock [3] The national reported incidence in 2012 was 31.1 per 100,000—a significant increase from previous years [4]. National statistics tend to underestimate the incidence of infectious gastrointestinal disease, as many cases are not notified, so the true incidence will be higher [5].

Infection is contracted via the faecal-oral route following ingestion or inhalation of environmentally resilient oocysts [6] either directly or indirectly through anthroponotic, zoonotic, and sapronotic sources [7]. Distinguishing characteristics that are critical to the epidemiology and spread of *Cryptosporidium* are as follows: (1) the lifecycle is completed within an individual host and oocysts are fully sporulated and infectious upon shedding; (2) the wide range of susceptible hosts, including humans; (3) oocysts are highly resistant in the environment, persisting for weeks to months after contamination and are not inactivated by chlorination; (4) as little as one to ten oocysts can establish infection in a susceptible host [6]; and infected calves shed as much as 3.89 × 10^10^ oocysts during a 6-day period of infection [8].

*Cryptosporidium* infections in humans are characterised by acute episodes of short-lived and self-limiting diarrhoea [9] and may be accompanied by abdominal pain, nausea, weight loss, fever, and headache, although asymptomatic infections are also common in animals and humans [10]. Symptoms usually resolve in one to two weeks, with oocysts being excreted for an additional one to four weeks. Chronic infections may occur due to the oocysts’ ability to excyst in the intestinal tract, and cause autoinfection [9]. Infection can lead to prolonged or life-threatening illness in immunocompromised patients [11]. The pathophysiological mechanism causing the diarrhoea is unclear, but is thought to be the result of direct mucosal damage by the parasite or an enterotoxin [12].

The two most commonly identified species affecting humans are *C. parvum* and *C. hominis*; the former infects all mammals and is endemic in calves [13], while the latter cycles primarily among humans and is transmitted anthroponotically. Zoonotic transmission is considered the most common route of infection of *C. parvum* for humans and is an important occupational risk for people working with livestock. Outbreaks of cryptosporidiosis have previously been reported among veterinary students as a result of direct or indirect contact with farm animals and through anthroponotic transmission [10,11,14,15,16]. These outbreak events highlight the need for stringent prevention and control procedures. An outbreak of cryptosporidiosis occurred at Massey University in 2006 with 25/80 (31%) respondents developing clinical signs from a cohort of 96 veterinary science students. Investigation at the time suggested that contact with apparently healthy calves during a practical class was the most likely exposure [17]. This paper describes the investigation of an outbreak of cryptosporidiosis among veterinary science and veterinary technology students at Massey University over an eight-week period during the spring-calving period in 2013.

## 2. Materials and Methods

### 2.1. Sequence of Events

MidCentral Public Health Service (MCPHS; the local district health board) received notification of a case of cryptosporidiosis in a veterinary student from Massey University (MU) on 4 September 2013. A further three notifications were received in the month of September. At this point the Coordinator of Health Protection MCPHS contacted staff at the veterinary faculty to discuss the situation and review the environment and hygiene procedures. MCPHS initiated an outbreak investigation and a questionnaire was developed for distribution to students who had been on clinical rosters after 19 August 2013 and may have been in contact with animals with diarrhoea. The investigation was a MCPHS investigation, thus human ethics approval was not sought. All veterinary science and veterinary technology students were advised of effective hygiene procedures to minimise the risk of cryptosporidiosis.

### 2.2. Case Definitions

A clinically-confirmed case was defined as a veterinary science or veterinary technology student from Massey University with diarrhoea (of at least 24 hours’ duration) and/or vomiting where the onset of illness was between 20 August 2013 and 10 October 2013. These dates bracketed the usual time period when calves with diarrhoea were brought into the university’s Veterinary Teaching Hospital for intensive treatment.

A laboratory-confirmed case was a clinically-confirmed case that tested positive for *Cryptosporidium*.

### 2.3. Questionnaires

The questionnaires were distributed to the second and third year Bachelor of Veterinary Technology (BVT; *n* = 56) and the final year (fifth) Bachelor of Veterinary Science students (BVSc; *n* = 100). The questionnaire was in five parts: (1) information about the respondent and the onset of disease; (2) the symptoms experienced, if any; (3) assessment of the risk of exposure via anthroponotic transmission; (4) assessment of the risk of zoonotic transmission by documenting clinical experiences and any contact with animals with diarrhoea; and (5) assessment of the risk of sapronotic transmission from outdoor activities that involved exposure to freshwater and the consumption of untreated drinking water. Students could indicate on the questionnaire that they consented to their anonymised responses being made available for data analysis.

Paper copies of the questionnaire were distributed directly to the students via their on-campus letterboxes on 7 October and a locked return box was provided on campus. The questionnaire was also sent electronically to the final year BVSc students, as many of them were undertaking external clinical placements at the time. An email reminder was sent a fortnight after the original distribution to remind all students to complete and return the questionnaire. The questionnaires were collected from the locked drop box on a number of occasions by a staff member from MCPHS to maintain confidentiality.

### 2.4. Microbiological and Molecular Analysis

Eight faecal specimens provided as part of the clinical investigation undertaken by the students’ own general medical practitioners were analysed for the presence of enteropathogens at a commercial human diagnostic laboratory. Enzyme-linked immunosorbent assays (ELISA) were used to detect *Campylobacter* (Premier Campy EIA; Meridian Bioscience Inc., Cincinnati, OH, USA) and *Giardia* and *Cryptosporidium* antigens in the eight faecal specimens (ProSpecT Giardia/Cryptosporidium EIA; Remel, Lenexa, KA, USA). Positive results were further tested on a rapid immunoassay (Immunocard STAT Giardia/Cryptosporidium; Meridian Bioscience Inc., Cincinnati, OH, USA) to distinguish between *Cryptosporidium* and *Giardia*.

One of the human faecal samples had genomic DNA extracted and genotyped at the Protozoal Research Unit, Molecular Epidemiology and Public Health Laboratory, at Massey University, as part of a New Zealand Ministry of Health contract. The faecal specimen was placed in the commercial NucleoSpin Soil kit (Macherey-Nagel GmbH & co. KG, Düren, Germany) and DNA was extracted as per the manufacturer’s instructions. The DNA was isolated for polymerase chain reaction (PCR) using Gp60 primers (Invitrogen, Auckland, New Zealand) for selective amplification of the Gp60 genetic marker [18]. The amplified PCR products were then sent for sequencing at the Massey Genome Service (Institute of Fundamental Science, Massey University: Palmerston North). The resulting sequence was edited and aligned using the software program Geneious (Biomatters, Auckland, New Zealand) and matched with already known genetic subtypes using the BLAST (Basic Local Alignment Search Tool) programme (National Library of Medicine, Bethesda, MD, USA).

Faecal specimens from 8 of the 31 calves with diarrhoea that were hospitalised at Massey University between 19 August and 7 October 2013 were submitted to New Zealand Veterinary Pathology (a commercial veterinary diagnostic facility) and were examined microscopically for the presence of *Cryptosporidium* oocysts after acid-fast staining [19] cultured for *Salmonella* and tested by ELISA for rotavirus and coronavirus.

### 2.5. Data Analysis

Questionnaire information was entered into an Excel spreadsheet (Microsoft Corporation, Auckland, New Zealand) and analysed using the software package R version 2.12.0 (R Foundation for Statistical Computing, Vienna, Austria). Age was categorised as 17–21, 22–24, 25+ years, and unknown, and the ethnicity of the students was categorised as European, Asian, and other. The data were also used to describe the natural history of the disease, produce an epidemic curve, estimate the attack rate, and perform a risk-factor analysis.

For the risk factor analysis, all of the students meeting the criteria for the definition of a laboratory or clinical confirmed case were considered cases. The strength of association between putative risk factors and the outcome binary variable (case or non-case) were initially assessed using univariable logistic regression analysis at the liberal *p* ≤ 0.2. Variables significant at *p* ≤ 0.2 were used to build a multivariable model using a backward selection method. Variables were retained if significant at *p* ≤ 0.05 or if their presence in the model changed a regression coefficient by more than 15%, thereby indicating confounding. In addition, the presence of interactions between age, ethnicity, and other independent variables were investigated after selecting variables for entry into the multivariable model.

## 3. Results

**Figure 1:**
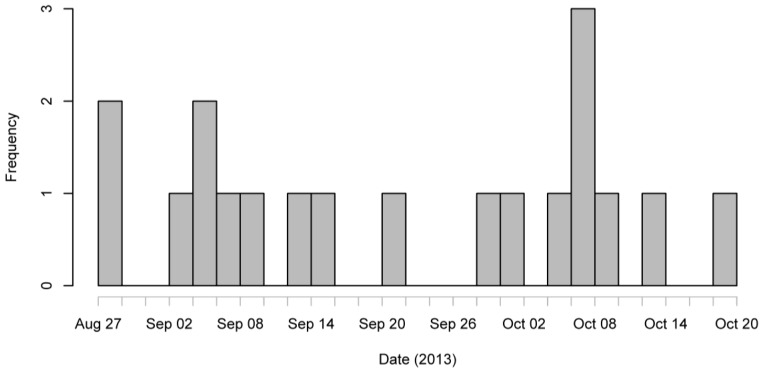
Epidemic curve of cases according to date of first symptoms among veterinary students during an outbreak of cryptosporidiosis.

A total of 80 questionnaires were returned between the 15 October and 4 November 2013 and an additional questionnaire was completed over the phone by MCPHS staff with a confirmed case. Thus, 81 questionnaires were returned. However, upon anonymising the questionnaires, it was noted that some sharing of the questionnaire had occurred between years of BVT students and 12 of the questionnaires completed were unexpectedly returned by BVT1 students. This makes the number of questionnaires returned from the 156 students that were deliberately distributed the questionnaire at 69 (BVT2, BVT3, and BVSc5), a response rate of 44.2%. Of the 81 questionnaires 74 were released for analysis, 52 were from non-cases. Of the remaining 22, three were unwell but did not fit the case definition as follows: one experienced stomach cramps only and the other two were overseas before and during the incubation period. Thus, nineteen respondents met the clinical case definition, one of whom was a BVT1 student, and seven of these were lab-confirmed cases. The attack rate was 19/81, 23% (95% CI 16%–34%). Symptoms experienced by the 19 cases included diarrhoea, vomiting, and nausea, and the prevalence of the symptoms are enumerated in Table 1. The epidemic curve (Figure 1) shows a continuous spread of cases from late August to mid-October. 

The demographic characteristics of the study and source populations stratified by program are reported in Table 2. One of the affected students was male (5%) and the remainder were female.

Of the 74 respondents, 51 (69%) had contact with at least one animal with diarrhoea during the study period. All students reported the species they had been in contact with, and 48 (94%) gave precise dates of when the contact with these animals occurred. The students most frequently reported exposure to calves with diarrhoea; the frequency of student exposure to animals with diarrhoea is shown in Table 3. From all questionnaires available for analysis, the average number of weeks in which the students were exposed to animals with diarrhoea over the 11-week period was 1.1 (median 1; range 0–4).

Three of the students defined as a case did not record contact with calves or other animals or people with diarrhoea prior to the onset of their clinical signs.

### 3.1. Microbiological Findings

Eight faecal specimens were submitted from individuals with gastrointestinal symptoms. Seven samples were positive for *Cryptosporidium* and the other faecal sample was negative for *Cryptosporidium* but positive for *Campylobacter*. One faecal specimen was subtyped as *Cryptosporidium parvum* belonging to the allelic group *IIa* A18G3R1. Over the time period 19 August to 7 October, 31 calves with diarrhoea were admitted to the large animal hospital at Massey University. There were also calves with other health problems, sheep, alpacas, and a pig that the students would have been in contact with. An aetiological diagnosis was not reached for all of the calves with diarrhoea due to cost constraints, the fact that it would make no difference to treatment protocol, and because most of these calves came from two farms and it was assumed that the causative agent would likely be the same in all calves from the same property. Faecal samples from eight calves were submitted to the local veterinary diagnostic laboratory for testing; a sample from one calf cultured *Salmonella* spp. and the remaining seven were found to contain *Cryptosporidium* oocysts, with four of these also being positive for rotavirus antigen. Table 4 shows the percentage of cases and distribution of those exposed to calves with diarrhoea during their studies.

### 3.2. Risk-Factor Analysis

Significant results at the liberal *p* value of ≤0.2 from the univariable screening are shown in Table 5. These variables were taken forward into the multivariable model. The only variable remaining significant at *p* ≤ 0.05 was exposure to calves with diarrhoea (Table 6). Contact with cows with diarrhoea was marginally significant (*p* = 0.07) and class remained in the model as a confounder. Students with one week of contact with calves with diarrhoea had approximately 10 times the odds of becoming a case of cryptosporidiosis (adjusted OR 10.61, 95% CI 1.87–108.29) than those unexposed to calves with diarrhoea. There was a dose response, with students with two weeks of contact with calves with diarrhoea having approximately 55 times the odds of becoming a case of cryptosporidiosis (adjusted OR 55.05, 95% CI 3.80–1931.18) in comparison to those unexposed to calves with diarrhoea. These estimates were adjusted for the effect of contact with cows with diarrhoea and class. None of the tested interactions were significant. 

### 3.3. Subset Analysis of Cases: Incubation Periods

Veterinary students handled 31 calves with diarrhoea in the large animal hospital at Massey University during the study period. Seven of thirty-one calves were confirmed to be shedding *Cryptosporidium*. Given a maximum incubation period of 12 days, there were three laboratory-confirmed cases of cryptosporidiosis among the students and a further seven clinically-confirmed cases who had contact with sick calves and onset of illness that would fit with the handling of these animals. Of these ten case students, eight did not have contact with any other animals with diarrhoea; one had contact with calves with diarrhoea at another location and became ill within the incubation period, and the other had contact with calves with diarrhoea at another location, but their onset of illness dates did not fit with the incubation period.

## 4. Discussion

An outbreak of gastrointestinal illness among Massey University’s veterinary science and veterinary technology students was strongly associated with exposure to calves with diarrhoea, some of which were confirmed to be due to *Cryptosporidium*. Seven of the students were confirmed as suffering from cryptosporidiosis. Furthermore, the subtype from the one human isolate that was genotyped, *C. parvum* subtype *IIa* A18G3R1, is the most common subtype in calves [20,21]. Other outbreaks of cryptosporidiosis among veterinary students have been linked to contact with calves [11,17]. The seasonal pattern of an increase of cases of cryptosporidiosis in humans in New Zealand coinciding with the calving and lambing seasons [22] provides further evidence that contact with neonatal livestock is a significant risk factor for disease in people. These findings constitute a significant occupational health and safety concern for people in contact with infected animals.

The risk of cryptosporidiosis in calves is increased when there is a higher density of animals and thus greater contamination of the environment [23,24,25]. Calves removed from their dams and reared in groups in confined spaces result in a much higher infection pressure for all enteropathogens and *Cryptosporidium* oocysts are particularly difficult to control with standard disinfection procedures. Seasonal calving, as occurs in New Zealand, means that large numbers of calves are reared over a short period of time and those calves born later in the season (and the people responsible for rearing them) can be exposed to a highly contaminated environment.

Awareness of risk from zoonotic disease is probably heightened when people are in contact with animals that are clinically affected with diarrhoea, however, even asymptomatic calves present a risk. A previous cryptosporidiosis outbreak among veterinary students at Massey University was associated with apparently healthy calves used during an animal handling practical class [17], so all people in contact with calves need to be aware of the risk of disease and the precautions that should be taken. Studies reporting the prevalence of cryptosporidiosis in calves commonly report the presence of oocysts in about 50% of calves in the 7–21 day age range [23,26] and lower prevalence in younger and older calves. However, when calves are repeatedly sampled during the pre-weaning period, a prevalence of 93% was reported [27].

Veterinary students at Massey University have been diagnosed with cryptosporidiosis in previous years. In fact, it is almost an annual occurrence during the calving season, despite ever-increasing hygiene measures and student education about zoonoses. Anecdotally, the attitude of veterinary students at Massey University towards zoonotic infections is changing. Previously, being affected by enteritis was seen almost as a rite of passage, whereas now students are more concerned with preventing infection. A variety of strategies have been implemented to reduce human cases of cryptosporidiosis including: demonstrations in class on the correct use of personal protective equipment (PPE), provision of case studies and personal stories of students who have experienced cryptosporidiosis which emphasise how unpleasant the symptoms can be, increased signage in the large animal hospital about the risks of zoonotic disease, more hand and boot-washing stations provided in the large animal hospital, and more hand sanitisers mounted within the veterinary teaching hospital. Most recently, gates have been installed in the farm animal corridor of the large animal hospital to further limit access to the patients, and sets of boots and waterproof bib-over-trousers that stay within the large animal hospital have been provided rather than students using their own PPE and attempting to disinfect it. All of these strategies can be implemented to minimise zoonotic transmission throughout veterinary education but are particularly important in the control of cryptosporidiosis due to the low infectious dose, the resistance of oocysts in the environment, and the risk of indirect contact with infectious material. The provision of keyboard covers in student computer laboratories has also been considered as a means of limiting transmission, but these would need to be regularly wiped down, as would door handles and other surfaces, so most effort has gone into preventing the movement of infectious material from the large animal hospital. Another approach to safeguard student health would be to limit the involvement that veterinary students have with sick calves, however, this approach must be balanced with the value of clinical experience in producing practice-ready new graduates.

The short delay between notification of the epidemic and onset of the investigation was a strength of this investigation and should have reduced recall bias in the questionnaire. The use of two separate case definitions, one for laboratory-confirmed cases, and one for clinically-confirmed cases, allowed the inclusion of students who may have had symptoms caused by other pathogens or non-infectious causes, such as the student who tested positive for *Campylobacter*. This overestimates the contribution of *C. parvum* to the burden of student disease but was worthwhile to capture possible cases, given that many of the students did not seek medical attention for their condition.

The identified relationship between class and the incidence of cryptosporidiosis showed a significant increased incidence of disease among the second year Bachelor of Veterinary Technology students (Table 5). A plausible explanation for this is that these students would not previously have had contact with sick animals from within the veterinary programme and therefore these students may be less experienced with barrier nursing skills and handling of patients with zoonotic disease. This finding suggests that instruction to this group of students could be more intensive and practical which would allow them the opportunity to acquire more skills by the time they have contact with possibly infected animals. Another factor which could have led to the increased incidence within this group would be that the population is likely to be naïve to *Cryptosporidium* infection due to the lack of prior exposure.

While this study has provided useful information about exposure to potential sources of infection with cryptosporidiosis among veterinary students, it contains potential limitations. Firstly, the study questionnaire only asked the respondents to list the animals with diarrhoea they had been in contact with and not the animals that appeared clinically normal. This is a limitation because it does not take into account the possibility of infection acquired from asymptomatic animals. Secondly, the incidence estimates may not be entirely representative of the total veterinary student population at Massey University, as enrolment in the study was voluntary.

In conclusion, an outbreak of gastrointestinal illness with some cases confirmed as cryptosporidiosis occurred among a cohort of Veterinary Technology and Veterinary Science students at Massey University. The infections were most likely transmitted from direct contact with young calves. The illness was self-limiting in all cases. This outbreak re-emphasizes the potential hazard for outbreaks of zoonotic cryptosporidiosis and the need for stringent control and hygiene procedures when handling young livestock.

## Figures and Tables

**Table 1:** Symptoms experienced by students who met the case definition during an outbreak of cryptosporidiosis in 2013.

Symptom	Number (%)
Diarrhoea	18 (95)
Vomiting	9 (4)
Stomach cramps	9 (4)
Other (nausea, fever, headache)	6 (32)

**Table 2:** Demographic characteristics of the students who met the case definition (*n* = 19), who consented to the release of questionnaire data (*n* = 74), and who were within the cohort (*n* = 187) during an outbreak of cryptosporidiosis in 2013. BVT1, 2, 3: first, second, and third year Bachelor of Veterinary Technology, respectively; BVsc5: fifth year Bachelor of Veterinary Science.

Variables	Students Who Met the Case Definition (%)	Questionnaires Available for Analysis (%)	Students within the Cohort (%)
Gender			
Male	1 (5)	4 (5)	27 (14)
Female	18 (95)	70 (95)	160 (86)
Ethnicity			
European	14 (74)	56 (76)	137 (73)
Asian	5 (26)	14 (19)	36 (19)
Other	0	4 (5)	14 (8)
Age			
17–21	4 (21)	17 (23)	23 (13)
22–24	7 (37)	31 (42)	83 (44)
25+	6 (32)	24 (32)	81 (43)
Unknown	2 (10)	2 (3)	0
Class			
BVT1	1 (5)	12 (16)	31 (17)
BVT2	7 (38)	11 (15)	26 (14)
BVT3	2 (11)	14 (19)	30 (16)
BVSc5	9 (46)	37 (50)	100 (53)
**Total**	**19 (100)**	**74 (100)**	**187 (100)**

**Table 3:** Students reporting exposure to animals with diarrhoea within each weekly roster during an outbreak of cryptosporidiosis in 2013.

Animal Type	Student Exposure to Animals with Diarrhoea (%)
Cow	8 (10)
Calves	42 (59)
Sheep and Lambs	6 (8)
Horses and Foals	5 (6)
Dogs	9 (12)
Cats	4 (5)

**Table 4:** Number of students by class that were cases, were exposed to calves with diarrhoea, and the corresponding proportion during an outbreak of cryptosporidiosis in 2013.

Class	Number of Students Exposed to Calves with Diarrhoea	Number of Students Defined as a Case	Proportion of Exposed Students that Became Cases
BVT1	1	1	1.0
BVT2	9	7	0.78
BVT3	8	2	0.25
BVSc5	24	9	0.38
Total	42	19	0.45

**Table 5:** Univariable analysis of putative risk factors associated (*p* ≤ 0.2) with being a case of cryptosporidiosis during an outbreak in 2013.

Variable		Number	Odds Ratio	95% CI	*p* value
Number of weeks of contact with calves with diarrhoea	0	32	REF		
	1	37	8.13	1.99–55.26	0.01
	2	5	60	5.88–1574.34	≤0.01
Number of weeks of contact with cows with diarrhoea	0	66	REF		
	1	8	3.4	0.73–16.02	0.11
Class *	BVT1	12	REF		
	BVT2	11	19.25	2.40–425.96	0.02
	BVT3	14	1.83	0.15–42.77	0.64
	BVSc5	37	3.54	0.56–69.15	0.26

* Program and year of the students; BVT = Bachelor of Veterinary Technology, BVSc = Bachelor of Veterinary Science.

**Table 6:** Multivariable analysis of putative risk factors associated with being a case of cryptosporidiosis during an outbreak in 2013.

Variable		Number	Odds Ratio	95% CI	*p* value
Number of weeks of contact with calves with diarrhoea	0	32	REF		
	1	37	10.61	1.87–108.29	0.02
	2	5	55.05	3.80–1931.18	0.01
Number of weeks of contact with cows with diarrhoea	0	66	REF		
	1	8	5.50	0.88–41.64	0.07
Class *	BVT1	12	REF		
	BVT2	11	2.96	0.18–81.00	0.45
	BVT3	14	0.28	0.01–9.34	0.44
	BVSc5	37	0.54	0.04–13.57	0.66

* Program and year of the students; BVT = Bachelor of Veterinary Technology, BVSc = Bachelor of Veterinary Science
